# Supplementary material for: Analysis of factors affecting visual comfort in hotel lobby
Source: PLoS One. 2023 Jan 19;18(1):e0280398. doi: 10.1371/journal.pone.0280398 (PMC9851505; doi:10.1371/journal.pone.0280398)
Supplement: S1 Appendix — (DOCX) [file pone.0280398.s001.docx]

Hotel lobby models


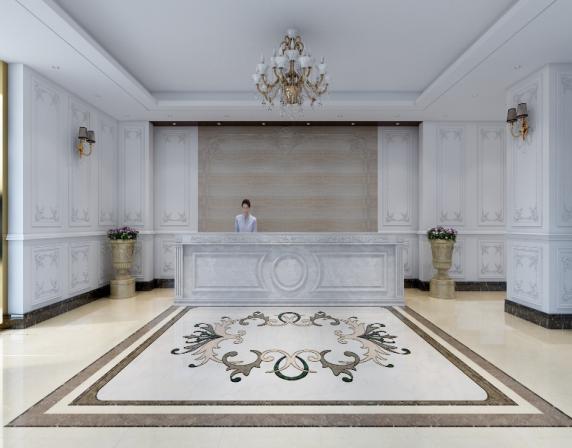

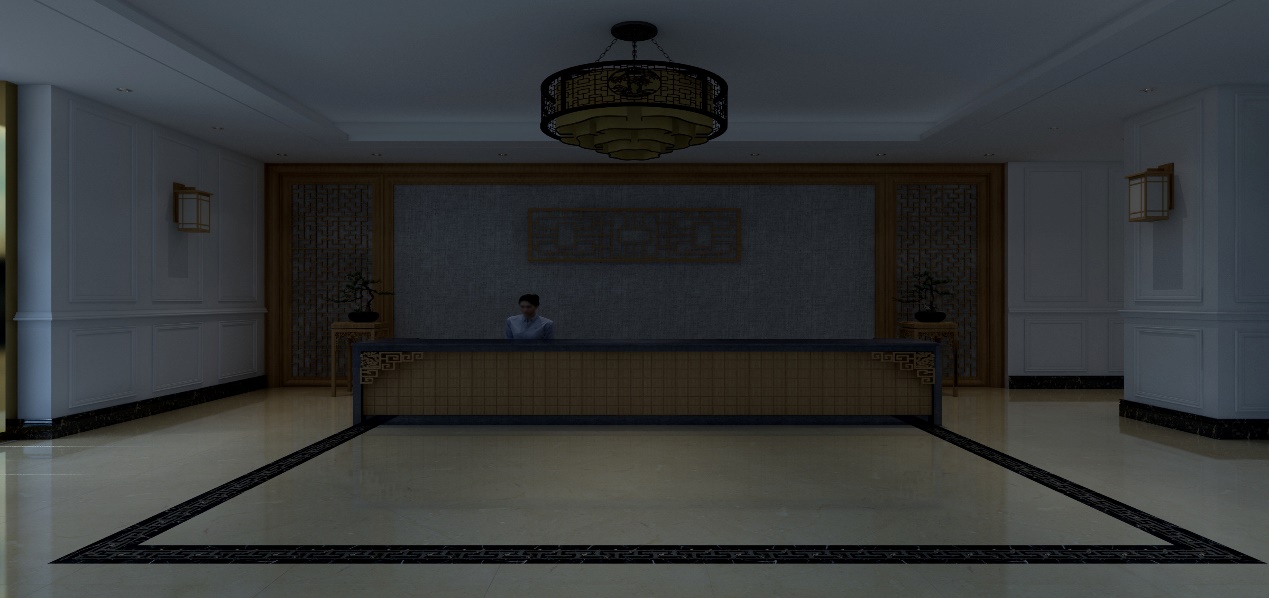

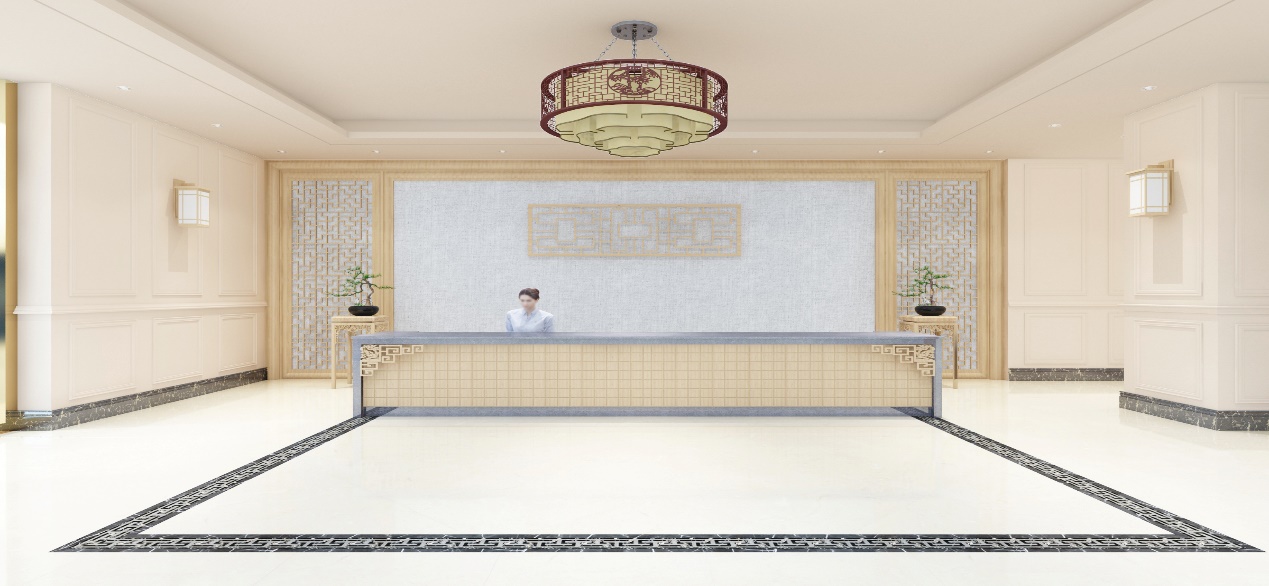


Model 1. European style normal illumination cool tone Model 2. Chinese style dim light and cool tone Model 3. Chinese style bright illumination warm tone


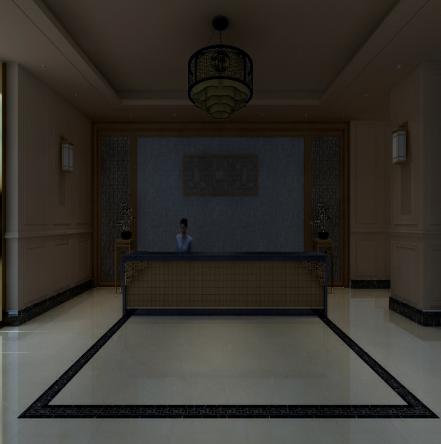

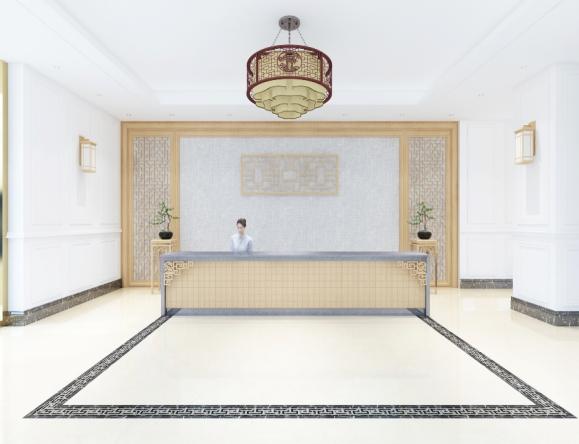

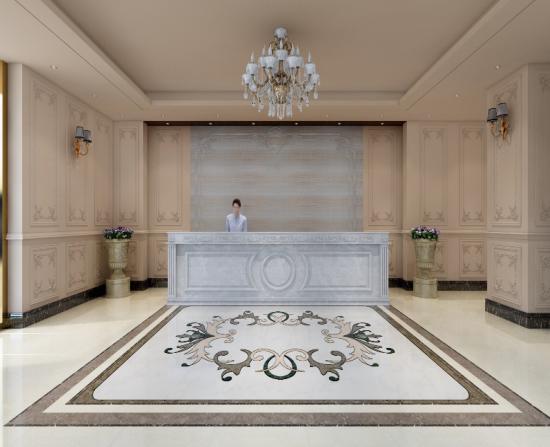


Model 4. Chinese style dim light and warm tone Model 5. Chinese style bright illumination cool tone Model 6. European style normal illumination warm tone


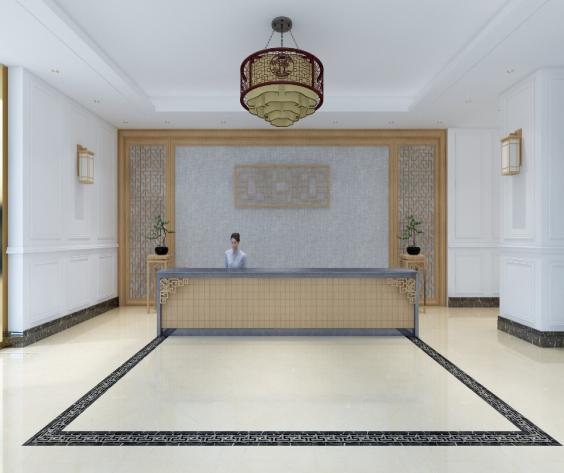

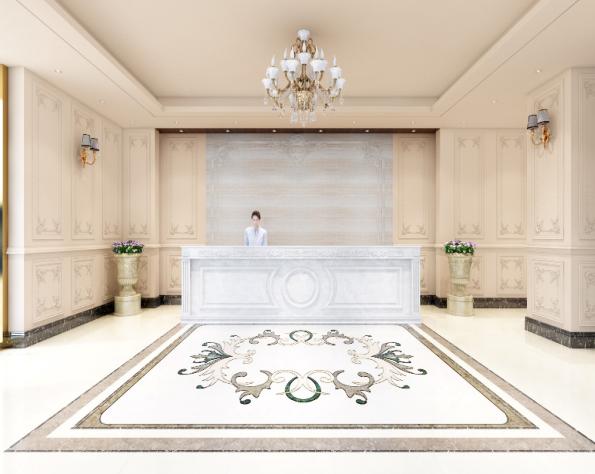

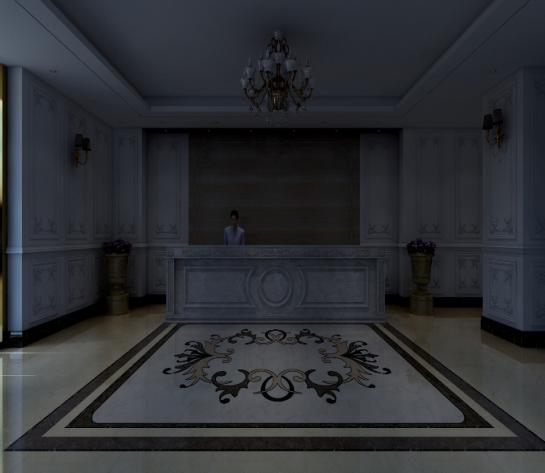


Model 7. Chinese style normal illumination cool tone Model 8. European style bright illumination warm tone Model 9. European style dim light and cool tone


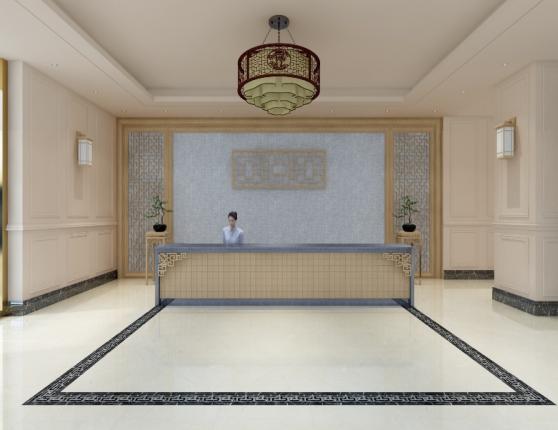

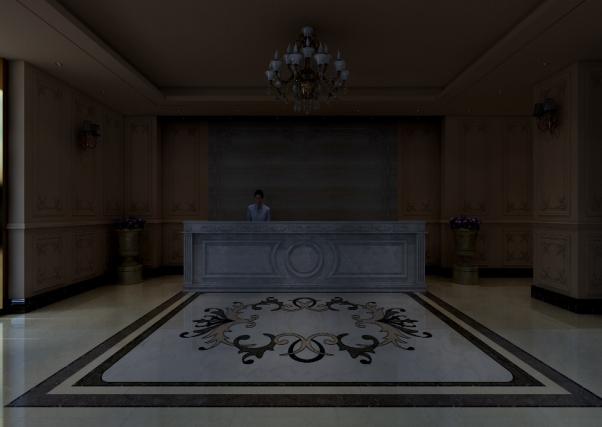

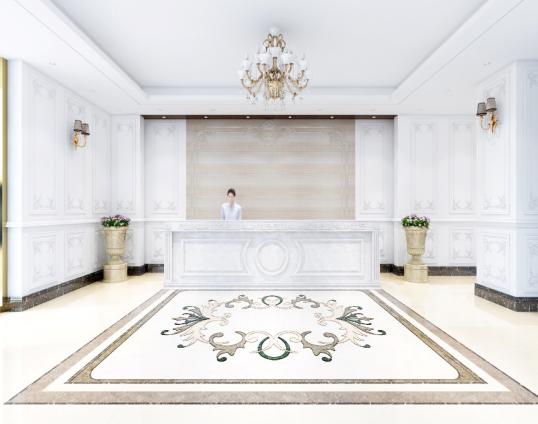


Model 10. Chinese style normal illumination warm tone Model 11. European style dim light and warm tone Model 12. European style bright illumination cool tone
