# Supplementary material for: Analysis of factors affecting visual comfort in hotel lobby
Source: PLoS One. 2023 Jan 19;18(1):e0280398. doi: 10.1371/journal.pone.0280398 (PMC9851505; doi:10.1371/journal.pone.0280398)
Supplement: S2 Appendix — (DOC) [file pone.0280398.s002.doc]

Hotel Visual Comfort Questionnaire

Dear friends:

Hello!

We are classmates of the Service Quality Research Group of China Jiliang University. At present, I want to conduct a survey on the quality of hotel services, in order to understand the current situation of hotel services and improve the quality of hotel services. Take a few minutes of your time to help fill out the questionnaire. This questionnaire is only for academic research and will not be made public. Please feel free to fill it out.

Please choose the option that best reflects your personal feelings based on your actual feelings during the experiment. Sincerely thank you for your cooperation and support!

**Basic information**

1. Gender

A. Male B. Female

2. Age

A. Less than 20 years old B. 21-30 years old C. 31-40 years old

D. 41-50 years old E. 51-60 years old F. 61 years old or older

3. Your education level

A. High school and below B. College C. undergraduate D. Graduate and above

4. Your occupation

A. business or management personnel B. government officials

C. self-employed D. professional and technical personnel

E. educators F. students G. other

1. The number of days staying in the hotel (the last check-in experience)

A. 1-3 days B. 4-5 days C. 5-7 days D. 7 days or more

6. Type of hotel staying (last staying experience)

A. Budget hotels B. Star-rated hotels C. Ordinary hotels D. Theme hotels

E. Chain Business Hotel F. Apartment Hotel G Homestay H. There are other ways to stay

**Experience information before check-in (the last check-in experience)**

1. Reasons for staying in the hotel
2. Tourism B.Visiting relatives

C.Official D.Others, please specify _____________________

2. Do you know that the source of this hotel’s information is?

A. The Internet B. Relatives and friends C. I have known it for a long time

D. Local people's introduction E. Mass media F. Books or related guides

G. Others, please indicate ______________________

3. What you value most when choosing a hotel is?

A. Location B. Price C. Surrounding environment D. Service E. Safety

F. Cleanliness G. Others, please indicate ______________________

4.Have you ever stayed in a Chinese-style or European-style hotel?

A.Yes B.No

5.Do you prefer Chinese-style hotels or European-style hotels?

A.Chinese-style hotels B.European-style hotels C.Both are the same, no preference

**Self-rating scale for emotion and visual comfort**

| Item | Score | Answer |
| --- | --- | --- |
| The overall visual comfort of the room | Very uncomfortable1—2—3—4—5—6—7 Very comfortable |  |
| The visual comfort brought by the intensity of the room light | Very uncomfortable1—2—3—4—5—6—7 Very comfortable |  |
| The visual comfort brought by the color of the wall of the room | Very uncomfortable1—2—3—4—5—6—7 Very comfortable |  |
| The visual comfort brought by the room decoration style | Very uncomfortable1—2—3—4—5—6—7 Very comfortable |  |
| Score based on your current emotions | | |
| Excited | Hardly 1—2—3—4—5—6—7 Very strong |  |
| Happy | Hardly 1—2—3—4—5—6—7 Very strong |  |
| Peaceful | Hardly 1—2—3—4—5—6—7 Very strong |  |
| Sadness | Hardly 1—2—3—4—5—6—7 Very strong |  |
| Anxiety | Hardly 1—2—3—4—5—6—7 Very strong |  |
| Nervous | Hardly 1—2—3—4—5—6—7 Very strong |  |
| Surprise | Hardly 1—2—3—4—5—6—7 Very strong |  |
| Disgust | Hardly 1—2—3—4—5—6—7 Very strong |  |
