# Supplementary material for: Analysis of factors affecting visual comfort in hotel lobby
Source: PLoS One. 2023 Jan 19;18(1):e0280398. doi: 10.1371/journal.pone.0280398 (PMC9851505; doi:10.1371/journal.pone.0280398)
Supplement: Attachment — Submitted filename: Response to Reviewers.doc [file pone.0280398.s004.doc]

**Response to Reviewer #1:**

Thank you very much for the insightful comments on our paper. We have revised our paper carefully according to these suggestions. In this note, we will first list your comments and then outline what we have done in response to these comments.

**Title**

**1 The title needs to be improved as the perspective of emotion was not well reflected in the study.**

**Response:** Thank you very much for your advice.

The emotion-based perspective in the title of this paper is mainly reflected in the measurement method. We use eye movement data to measure comfort for three reasons.

(1) Visual comfort can be defined as “subjective conditions of visual happiness caused by the visual environment”(EN 12665, 2002). A large amount of psychological evidence shows that visual comfort and emotion are inextricably linked. For example, in a visually comfortable environment, people will feel relaxed, happy and excited(Corlett E N,1976). Some scholars’ comfort assessment questionnaire is to ask whether the subjects are happy (Yoon et al., 2014).
(2) Using eye movement data to measure emotion to characterize comfort. There have been many mature studies that use eye movement indicators to measure emotions. For example, Peter Walla et al.(2011) used indicators such as blink amplitude to measure consumer sentiment and brand preference(Peter Walla et al.,2011). Jingwen Yang et al.(2022) used fixation time and pupil diameter to measure the relationship between subjects’ facial emotion processing characteristics and speech communication ability (Jingwen Yang et al., 2022). Faye Knickerbocker et al. used eye tracking to explore the processing of emotional words(Faye Knickerbocker et al., 2019). Geraets used eye tracking to help identify real-world facial emotion(Geraets et al.,2021), etc.
(3) Research by Vytautas Abromavičius and Artūras Serackis(2018) had shown that pupil diameter and the number of fixation points can be used to measure visual comfort. The “uncomfortable” visual comfort score had the highest pupil size and lower number of fixation points(Vytautas Abromavičius and Artūras Serackis, 2018). We modified as your suggestion. We changed the title to Analysis of Factors Affecting Visual Comfort in Hotel Lobby.

**Introduction**

**1: what’s the main rational for integrating the visual elements with auditory stimulus when to measure visual experience? As the author mentioned there were many factors influencing the visual comfort the author proposed the visual element that function as a more important role, such as illuminance, color and decoration style. The auditory stimulus “music” was also involved, how does it influence visual comfort?**

**Response:** Thank you very much for your comments. There are interactions between the senses. The tone stimulus can alter the pleasantness rating of stimuli from other senses(Dematte`et al., 2007; Pollatos et al. 2007; Logeswaran and Bhattacharya, 2009). For example, congruent sounds can enhance odor pleasantness to a higher degree than can incongruent sounds(Seo H S, 2011). The interaction between senses was mainly reflected in their direct impact on emotions(Kantonoet al., 2019). Although lighting, color, and decoration style were all key visual factors, the hotel lobby often brought the overall sensory stimulation to the customer. For example, music generally interacted with other environmental factors and music emotion was significantly correlated with lighting(Hsiao et al., 2017; Morinet et al., 2007). Music genres influenced consumer lighting preferences. There were also structural correspondences between color and music (such as emotional expression, hierarchical organization and contrast)(Hsiao S W, 2017).

Music enhances the overall quality of the service landscape, and a background with music can increase positive consumer evaluations compared to an environment without music (Morin S, 2007). Demoulin (2011) studied the consistency of music and the overall atmosphere, and the results showed that when coordinated with the service environment and music, the relaxing, calm and pleasant atmosphere would be created. Pleasure induced by music congruency increases customers’ evaluation of environment quality and service quality, improves the customers’ perception of service quality(Demoulin, 2011).

The conclusions drawn from the eye-tracking data are consistent with the conclusions drawn by Hsiao(2017) that music affects consumers’ lighting as well as color visual comfort. However, compared with the background non-consistent music style, the music style consistent with the environment does not significantly enhance the visual comfort of consumers. The conclusion drawn from the questionnaire data is that music does not affect consumers’ visual comfort, and there is no interaction between music style and lighting, color and decoration style. The differences between the two measurement methods were analyzed as follows: Sebba’s research had confirmed that there was a structural correspondence between color and music(Sebba R, 1991). Since this reaction was unconscious and caused a relatively small degree of emotional change, it was only through subjective judgment and the real mood changes of the subjects cannot be accurately measured, but such changes can be significantly reflected in the eye movement indicators.

**2 What the definition of “Visual comfort” ? if people stand in a hotel lobby, their feelings would be affected with visual elements but the visual comfort was not the whole story. I guess the author aims to measure the psychological and physiological comfort. However, the approach employed was mainly influence the visual experiences as only the pictures but not the real physical stimuli were exposed. In other word, it would be flexible to test these effects by using the virtual reality display technology and the current study paradigm largely influence visual comfort and perception.**

**Response:** Thank you very much for your comments. Visual comfort can be defined as “subjective conditions of visual happiness caused by the visual environment”(EN 12665, 2002). In a visually comfortable environment, people would feel relaxed, happy and excited(Corlett, 1976). Comfort was a state in which people and the environment were relatively balanced in psychology, physiology and physics. When the human body felt balanced, it was called comfort. If this balance was broken by external factors, it would produce an uncomfortable feeling(Corlett, 2012). In this paper, visual comfort is defined as the physiological and psychological pleasure balance caused by external visual stimuli such as lighting and color.

Relevant statistics showed that more than 80% of the information acquired by the human body came from vision, and vision was the most important perception of humans and animals. Diţoiu and Cǎruntu(2014)interviewed 500 young people and found that when they selected destinations, visual elements accounted for 98.2% of the perceptual evaluation factors(Diţoiu and Cǎruntu, 2014). So we focused on the impact of lighting, wall color, decoration style and music on visual comfort.

Due to the limitations of eye trackers, this study can only be carried out in the laboratory, allowing subjects to see the 3D model of the hotel but not the real physical stimuli, which is a limitation of this study and needs to be improved in follow-up studies.

**3. For each visual elements, in addition to light level, the spectrum such as warm light or cool light would also be a factor influencing visual experience, the author also stated it in the introduction, but it was not involved in manipulation of the light of the model.**

**Response:** Thank you very much for your comment. In addition to the variables involved in this study, there are many other visual influencing factors. In the final limitations of the article, we mentioned that this study also had certain limitations, this paper only conducted preliminary research on the four influencing factors of visual comfort in the hotel lobby. In fact, the visual comfort of the hotel lobby would be affected by other factors, such as the layout of the lobby, the size of the space, the color of the lights, etc. In the future work, we will further investigate the effects of these variables on consumers’ visual comfort.

Light intensity is the three indicators determined according to the feelings of the subjects in the preliminary experiment. The bright intensity is 13000cd, the normal intensity is 9000cd, and the dim intensity is 2000cd. The data in Table1 is an indicator of the color of the wall, which does not involve lighting, and the text has been modified.

4. **What the decoration style was employed for Chinese and European style？The decoration style itself was indeed created with including multiple visual elements, such as light level, color and furniture etc. that would also moderate the visual experience, how did the author deal with that when to manipulate the experimental conditions?**

**Response:** Thank you very much for your comments. Picture1 is a Chinese style hotel lobby model with normal illuminance and cool color tone, and picture2 is a European style hotel lobby model with normal illuminance and cold color tone. Twelve hotel lobby models have been uploaded as attachments.

The decoration style itself is created with including multiple visual elements, such as light level, color and furniture etc., that will also moderate the visual experience. Firstly, we observe a large number of hotel lobby cases, and then find a decoration design expert to help us design the model diagram, retain the basic elements of the lobby, and control other variables. Finally, after expert evaluation and the opinions of the pre-experiment subjects, the model is continuously adjusted, and finally the Chinese-style and European-style hotel lobby models are designed.

On the one hand, when designing the hotel lobby model, we keep the basic elements of the lobby and control other variables unchanged. Twelve 3D lobby models are made by 3d Max software according to three variables: 3 (light intensity: bright, normal, dim) ×2 (wall color: cool color, warm color) ×2 (decoration style: European style, Chinese style). On the other hand, in the formal experiment, the experiment is carried out in an eye-tracking laboratory with a constant temperature of 25°, and the lights illuminate the entire space evenly with constant illumination. All subjects perform the experiment in the same environment.

Fig.1. European style normal illumination cool tone hotel lobby model

Fig.2. Chinese style normal illumination cool tone hotel lobby model

5. **Generally, the literature review on the effect of visual elements on comfort and mood need to be improved, and what the effects already have been investigated and what need to be explored? Especially, how these factors interactionally influenced visual experiences that the authors hypothesized more subjectively.**

**Response:** Thank you very much for your advice. We have refined a literature review of the effects of visual elements on comfort and mood.

(1)Color, emotion and visual comfort in service setting

In an experimental study, some scholars studied the differences between two contrasting hues (blue and red), as well as two levels of saturation and brightness. The results indicated participants enjoyed staying in blue hotel rooms more than in the red ones. The blue room was more pleasant, comfortable, and relaxing(Katsiaryna Siamionava et al., 2018). In the hotelscape, color had been shown to significantly impact customers’ aesthetic perceptions(Alfakhri et al., 2018). A comparison of warm and cool colors revealed that cool colors were not aesthetically superior to warm colors in the luxury hotel context and warmer colors were more pleasing(Warakul Tantanatewin and Vorapat Inkarojrit, 2018; Dongyoup Kim et al., 2020). However, an academic study also demonstrated no statistically significant effects of color and temperature on comfort results within the tested settings(Arianna Latini et al., 2021).

It can be seen that the influence of color on consumer comfort is different in different environments, but most studies have concluded that warm colors have higher visual comfort.

(2)Light illuminance, emotion and visual comfort in service setting

Lighting was an indispensable factor in the design of the hotel lobby. Lighting affected the overall impression and perceived comfort of the lobby(Akita, 2019; Countryman and Jang, 2006). The level of visual comfort in lighting in a residential context turned to be more influenced by the color temperature and illuminance compared to other factors（Yoon et al., 2014）. Under high illumination, the level of anxiety would decrease, which was more conducive to relieving fatigue. Providing a user-friendly and user-controlled lighting system for hotel rooms helped improve customer satisfaction(Akita, 2019). Light not only affected people’s emotions, but the interaction between light and sound also affected people’s collection of target information. Low-illumination light was more likely to cause visual fatigue. Lower contrast colors with darker lighting and simple decoration made it easier to create a relaxed and romantic atmosphere. Dim light with soothing music can create a relaxed and comfortable atmosphere(Siamionava et al., 2018). A lower color temperature would create a higher sense of security, positive feelings and restfulness(Akita, 2019). While some scholars found that brighter lights were more conducive to positive emotions(Figueiro, 2016).

It can be seen that high illumination is conducive to relieving fatigue, and low illumination is conducive to creating a relaxed and pleasant atmosphere. Different environments have different requirements for lighting illumination, so the normal illumination between the two may be more suitable for the needs of the general public.

(3)Color, light illuminance, music, emotion and visual comfort in service setting

There were interactions between the senses, the interaction between senses was mainly reflected in their direct impact on emotions(Kantonoet et al., 2019). The tone stimulus can alter the pleasantness rating of stimuli from other senses(Dematte`et al., 2007; Pollatos et al. 2007; Logeswaran and Bhattacharya, 2009). Music generally interacted with other environmental factors and music emotion was significantly correlated with lighting(Morin et al., 2007; Hsiaoet et al., 2017). There were also structural correspondences between color and music (such as emotional expression, hierarchical organization and contrast)(Sebba, 1991). Demoulin(2011) studied the consistency of music and the overall atmosphere, and the results showed that when coordinated with the service environment and music, the relaxing, calm and pleasant atmosphere would be created(Demoulin, 2011).

Although music is not a visual factor, it will have an impact on the visual comfort of lighting, color and style. There was an interaction between these four factors. Previous scholars’ research is more on the interaction of color and lighting, music and lighting or interaction of colors. What we see in real life is the overall comfort of the hotel lobby, not the comfort brought by individual lights or colors. Therefore, these four factors are inseparable, and we put them in a model to study.

**6.The main measurement of the current study was the visual comfort. The author stated a lot on measurement of mood, please rephased it to be more concise and also I missing how the main visual elements influence mood in previous literature review.**

**Response:** Thank you very much for your advice. To update the literature, we repositioned the mood and comfort measures to be more concise. We used eye movement data to measure comfort for three reasons.

The definition of comfort was that consumers were in a state of pleasure and peace(Corlett, 1976). Some scholars’ comfort assessment questionnaire was to ask whether the subjects were happy(Yoon et al., 2014).

Using eye movement data to measure emotion to characterize comfort. There have been many mature studies that use eye movement indicators to measure emotions. For example, Peter Walla et al.(2011) used indicators such as blink amplitude to measure consumer sentiment and brand preference(Peter Walla et al., 2011). Jingwen Yang et al.(2022) used fixation time and pupil diameter to measure the relationship between subjects’ facial emotion processing characteristics and speech communication ability(Jingwen Yang et al., 2022). Faye Knickerbocker et al.(2019) used eye tracking to explore the processing of emotional words(Faye Knickerbocker et al.,2019). Geraets(2021) used eye tracking to help identify real-world facial emotion(Geraets et al.,2021), etc.

Research by Vytautas Abromavičius and Artūras Serackis(2018) had shown that pupil diameter and the number of fixation points could be used to measure visual comfort. The “uncomfortable” visual comfort score had the highest pupil size and lower number of fixation points.(Vytautas Abromavičius and Artūras Serackis, 2018).

A review of the research on the effects of lighting and color on mood and comfort is as follows, in the Introduction chapter of the paper.

(1)Color, emotion and visual comfort in service setting

Color is one of the key visual dimensions of the environment that has an impact on behavior and emotions. The emotional effect of color was particularly important in the hospitality industry because emotions comprise a powerful affective component of customer satisfaction. The differences between two contrast hues (blue and red) with two levels for saturation and brightness were examined in an experimental study. The results indicated participants enjoyed staying in blue hotel rooms more than in the red ones. The blue room was more pleasant, comfortable, and relaxing（Katsiaryna Siamionava et al., 2018）.In the hotelscape, color had been shown to significantly impact customers’ aesthetic perceptions (Alfakhri et al., 2018). A comparison of warm and cool colors revealed that cool colors were not aesthetically superior to warm colors in the luxury hotel context and warmer colors were more pleasing(Warakul Tantanatewin and Vorapat Inkarojrit , 2018; Dongyoup Kimet et al., 2020). However, an academic study also demonstrated no statistically significant effects of color and temperature on comfort results within the tested settings(Arianna Latiniet et al., 2021).

(2)Light illuminance, emotion and visual comfort in service setting

Lighting was an indispensable factor in the design of the hotel lobby. Lighting affected the overall impression and perceived comfort of the lobby(Akita, 2019; Countryman and Jang, 2006). The level of visual comfort in lighting in a residential context turned to be more influenced by the color temperature and illuminance compared to other factors(Yoon et al., 2014). Under high illumination, the level of anxiety would decrease, which was more conducive to relieving fatigue. Providing a user-friendly and user-controlled lighting system for hotel rooms helped improve customer satisfaction(Akita, 2019). Light not only affected people’s emotions, but the interaction between light and sound also affected people’s collection of target information. Low-illumination light was more likely to cause visual fatigue. Lower contrast colors with darker lighting and simple decoration made it easier to create a relaxed and romantic atmosphere. Dim light with soothing music can create a relaxed and comfortable atmosphere(Siamionava al., 2018). A lower color temperature would create a higher sense of security, positive feelings and restfulness(Akita, 2019). While some scholars found that brighter lights were more conducive to positive emotions(Figueiro, 2016). Different environments have different lighting requirements.

(3)Color, light illuminance, music, emotion and visual comfort in service setting

There were interactions between the senses, the interaction between senses was mainly reflected in their direct impact on emotions(Kantonoet et al., 2019). The tone stimulus can alter the pleasantness rating of stimuli from other senses(Dematte`et al., 2007; Pollatos et al., 2007; Logeswaran and Bhattacharya, 2009). For example, congruent sounds can enhance odor pleasantness to a higher degree than can incongruent sounds(Seo H S, 2011). Music generally interacted with other environmental factors and music emotion was significantly correlated with lighting(Morin et al., 2007; Hsiao et al., 2017). There were also structural correspondences between color and music(such as emotional expression, hierarchical organization and contrast)(Sebba, 1991). Demoulin (2011) studied the consistency of music and the overall atmosphere, and the results showed that when coordinated with the service environment and music, the relaxing, calm and pleasant atmosphere would be created(Demoulin, 2011).

**7. The hypothesis could be given at the end of the introduction to make the literature review more continuously.**

**Response:** Thank you very much for your advice. We have made changes to give hypothesis at the end of the introduction. We have reorganized the content of the introduction and literature review, and integrated the literature review into the introduction, please refer to it.

8. **The reference style presented in the introduction as well as elsewhere need to be improved by reference management tool with the requirement of the journal.**

**Response:** Thank you very much for your advice. Reference styles in the introduction and elsewhere have been improved according to journal requirements through reference management tools, please check it.

**Method**

**1.How was the sample size decided? Did you employed a G-power analysis to see how many participants were required with the current design?**

**Response:** Thank you very much for your comments. Physiological experiments are generally effective for subjects requiring more than 20 people, so this study did not use G-power analysis to determine the number of subjects. We recruited subjects directly and eventually recruited 43 valid subjects. Evidence of the feasibility of our approach was as follows, for example, Peter Walla et al.(2011) used an eye-tracking experiment to study emotional and physiological responses to brand attitudes in 29 subjects (Peter Walla et al., 2011). Research by Vytautas Abromavičius and Artūras Serackis showed that pupil diameter and the number of fixation points can be used to measure visual comfort. The “uncomfortable” visual comfort score had the highest pupil size and lower fixation points. There were 24 subjects in the eye movement experiment(Vytautas Abromavičius and Artūras Serackis, 2018). Borisuit et al.(2014) showed that workplace lighting conditions affected various factors related to job satisfaction, productivity, and well-being. Lighting conditions affected visual comfort, mood, alertness, and well-being. 25 People were tested(Borisuitet et al. 2014). Cajochen et al.(2019) studied that lighting conditions affected subjects’ visual comfort, mood, and sleep intensity. The number of subjects was 15(Cajochen, 2019).

**2.The participants had hotel experiences but did they had preferences to decoration style that might be a response bias in evaluating the experimental stimulus.**

**Response:** Thank you very much for your comment. The subjects of the pre-experiment have experience of staying in Chinese-style and European-style hotels, the subjects of the formal experiment have the experience of staying in ordinary hotels, but have no experience of staying in Chinese-style or European-style hotels. They have no apparent preference for style. We have added this part to the Participants module in the text.

**3.What’s the study design? Within or between-subjects design and how the pictures were arranged for each participant? Was it randomized or presented with a fixed order?**

**Response:** Thank you very much for your comments. The experiment adopted a within-subject experimental design. Each participant looked at 5 sets of model pictures (1. Observing the 12 hotel lobby models without music. 2. Observing the 6 Chinese-style lobby models in which Chinese music was played. 3. Observing the 6 Chinese-style lobby models in which European music was played. 4 . Observing the 6 European-style lobby models in which Chinese music was played. 5. Observing the 6 European-style lobby models in which European music was played.) . Each group of experimental data collection consisted of two parts.

Before the experiment, Part 1, let the subjects familiarize themselves with the laboratory environment and experimental requirements firstly, and then let the subjects sit in a comfortable chair. Set the distance between the eyes and the screen to 60 cm. When the five-point gaze tracking correction reached the standard level, the formal experiment began. During the formal experiment, participants would see detailed experimental instructions on the screen, and the presentation time is 5s (“You are a consumer who is preparing to stay at the hotel. Then you will walk into the hotel lobby and see the lobby environment. The lobby model will be automatically presented after 7s.”), a black screen for 2 seconds appeared, followed by stimulus images (the presentation time of each stimulus image was 5s, and a 2s black screen appeared between alternate images).The second part, the stimulus picture was presented again, and the participants verbally explained their emotions and comfort experience to the experimenters. After offering the answer, the subjects rested 10 minutes for the next set of experiments. The hotel lobby model diagram is randomly presented.

**4.The experiment was approved by the relevant department, what’s the department? Was there a ethical statement as human participants were involved?**

**Response:** Thank you very much for your comments. This experiment was approved by the Review Board of China Jiliang University.

There was an ethics statement and each participant gave written informed consent. This part of the content has been perfected in the paper.

**5.What the 3D pictures look like? Did them matched with brightness, familiarity etc. that would be potential factors influencing visual experiences and affective state? Also, was there only one sample for each kind of picture with manipulating illuminance, color and decretory style?**

**Response:** Thank you very much for your comments. Twelve 3D lobby models were made by 3d Max software according to three variables: 3 (light illuminance: bright illuminance, normal illuminance, dim illuminance) ×2 (wall color: cool color, warm color) ×2 (decoration style: European style, Chinese style).Twelve hotel lobby models have been uploaded as attachments.

After repeating adjustments based on the pre-experiment subjects and the opinions of experts, the model diagram was finalized. The model map was matched to the potential familiarity of the subjects.

There is only one sample for each kind of picture, and only one lighting level, one wall tone and one decoration style for each model picture. The 12 model diagram types are shown in Figure 1.

Fig.1.Hotel lobby model

**6.The hall model employed in the pre-experiment were same with that in the current experiment? What the aim of the pre-experiment? To verify the questionnaires? Did participants assessed the illuminance, color and decretory style in the pre-experiment? What the differences or adjustment had been done for formal experiment? Please state that in the method section.**

**Response:** Thank you very much for your comments.

The hall models employed in the pre-experiment were same with those in the current experiment.

The pre-experiment has the following two purposes: 1. To test the scientificity and feasibility of the experimental design. 2. To assess the feasibility of the questionnaire, whether the question description is clear, etc.

Before the pre-experiment, when designing the hotel lobby model, we invited participants to evaluate whether the lighting illumination, wall color and decoration style of the hotel lobby model meet the cognitive standards, and whether the hotel lobby design is reasonable. According to the opinions of the participants, we adjusted the hotel lobby model and added a service person behind the counter. Reducing the light intensity of normal lighting and dim lighting in the lobby.

Thank you very much for your suggestion. We modified as your suggestion. We have added this part to the method section.

**7.Please rephrase the statement of the procedure as what had been done before, during and after the experiment? Also the five sets of experiment seem to be five sessios with each session including two blocks. It’s confusing that the experiment consisted five sets of experiment.**

**Response:** Thank you very much for your suggestion. We have re-planned the experimental flow chart, and the statement of the procedure has been re-described as what was done before, during and after the experiment.

To investigate the influence of musical style on the other three visual factors, we designed five sets of experiments.

In the first group, under no music background, the subjects observed the 12 hotel lobby models (The first group of experiments collected two parts of data. 1. Collecting eye movement data, 12 hotel lobby models appeared randomly, and eye trackers captured the eye movement data. 2. Collecting the questionnaire data, and the subjects will score on the questionnaire after watching the model).

The second group, under the background of European-style music, the subjects observed the European-style hotel lobby (The second group of experiments collected two parts of data. 1. Collecting eye movement data, 6 hotel lobby models appeared randomly, and the eye tracker captured the eye movement data. 2. Collecting the questionnaire data, and the subjects will score on the questionnaire after watching the model).

The third group, under the background of European music, the subjects observed the Chinese-style hotel lobby (the third group of experiments collected two parts of data. 1. Collecting eye movement data, 6 hotel lobby models appeared randomly, and the eye tracker captured the eye movement data. 2. The questionnaire data was collected, and the subjects scored on the questionnaire after watching the model).

The fourth group, under the background of Chinese music, the subjects observed the Chinese hotel lobby (the fourth group of experiments collected two parts of data. 1. Collecting eye movement data, 6 hotel lobby models appeared randomly, and the eye tracker captured the eye movement data. 2. The questionnaire data was collected, and the subjects scored on the questionnaire after watching the model).

The fifth group, under the background of Chinese music, the subjects observed the lobby of a European-style hotel (the fifth group of experiments collected two parts of data. 1. Collecting eye movement data, 6 hotel lobby models appeared randomly, and the eye tracker captured the eye movement data. 2. The questionnaire data was collected, and the subjects scored on the questionnaire after watching the model).

Fig.2. Flow chart of experimental stimulus presentation

**8.What’s the 3D picture looks like? Please give samples, probably in the supplementary file.**

**Response:** Thank you very much for your comment. The 12 model diagram types are shown in Figure 3.

Thank you very much for your suggestion. We have added the hotel lobby model diagrams to the supplementary file.

Fig.3.Hotel lobby model

**9.The full name of the PANAS needs to be give where it first occurred.**

**Response:** Thank you very much for your suggestion. When PANAS first occurred, we gave it its full name.

**10.How participants assessed their comfort? By using standard questionnaire and what’s the response?**

**Response:** Thank you very much for your comments. There are two ways to measure comfort. First, it is measured by the comfort scale and scored using a 7-point Likert scale. The second is to reflect comfort through eye movement indicators. The subjective scale and physiological index data are comparative analyzed. The research conclusion is consistent with that of Corlett and Bishop(Corlett and Bishop, 2012). Consumers will be in a pleasant and peaceful emotional state when they are in a state of visual comfort. The comfort measurement questionnaire is uploaded as an attachment.

**11.What the interested indicators of the eye movement recording and what did they mean? As the reflection of visual comfort or mood？please state it in the method as well as in the introduction.**

**Response:** Thank you very much for your comments. The eye movement indicators used to reflect mood and visual comfort are pupil diameter and fixation points. The pupil diameter is used to describe the size of the pupil. The pupil diameter is not only affected by the surrounding environment, but also by the person's mood and interest in the target. The fixation point is the stop point of the eye on the observation target. Usually, when the duration of these stay points is more than 100ms, it is called fixation, and the number of fixation points is the number of these staying points (Duchowski, 2003).

In this study, pupil diameter and fixation number were used to reflect mood and visual comfort. Thank you very much for your suggestion. We have added this section to the Methods and Literature review.

**12.What the statistical method and tool employed for data analysis was missing?**

**Response:** Thank you very much for your comment. The tool used for data analysis is R3.5.0, and the method used is analysis of variance. They are described in the process and results section of the paper, please check it.

**Results**

**1.The structure of the results section for eye movement indicators as well as for the subjective indicators needs to be adjusted. As for main factors except for illuminance, only two levels were involved, the post-hoc contrasts did not need. Please added the relationship after the corresponding main effect presented. For interaction, please added the statement of the post-hoc contrast following the corresponding interaction effect. It would be clearer to separate main effects and interaction effects into two paragraphs. If the post-contrast were employed, the Bonferroni correction need to be applied.**

**Response:** Thank you very much for your advice. We have adjusted for the structure of the outcome sections for eye movement and subjective measures, applying a Bonferroni correction for multiple comparisons for post hoc comparisons. We divided the main effect and the interaction effect into two sections, only performed a post-event comparison of the light illuminance, and presented the post-event comparison results after the main effect. For interactions, the post-event comparison results are also added to the corresponding interaction effects.

**2.What was the analysis method? LMM model or ANOVA? Was the df presented in the table?**

**Response:** Thank you very much for your comments. The analysis method is ANOVA, and df was presented in the table of data analysis results. Please check it.

**3.Please correct the front style of statistical symbols, P should be p and all symbols need to be in italic. If the exact p values were given, the significant level did need any more.**

**Response:** Thank you very much for your advice. We have corrected the front style of statistical symbols, P to p and all symbols to italics.

We provide P-values according to journal requirements, Report exact p-values for all values greater than or equal to 0.001. P-values less than 0.001 may be expressed as p < 0.001

**4.Also the statistics were presented in the main text, the value resented in the table was redundant. It would be better to added the descriptive for each indicator in the table.**

**Response:** Thank you very much for your advice. We have removed numerical descriptions in the text that duplicated the table. We have added descriptions of indicators such as MSE and df. Indicators such as light illuminance are described in detail in the article Apparatus and stimuli, which will not be repeated here.

**5. Please remove the statements regarding the hypothesis in the results section, it would be redundant and can be added in the discussion.**

**Response:** Thank you very much for your advice. We have removed the statement of assumptions in the Results section and added this section to the Discussion.

**Discussion**

**1.The discussion needs to be rephrased and improved as what the main finding of the current study?**

**Response:** Thank you very much for your advice. Based on the main findings of the study, we have rephased and refined the discussion. Please check it.

**2.What the newly effects were revealed in the current study, were it the same or different from what the finding reported in previous studies and why?**

**Response:** Thank you very much for your comment.

The experimental results show that there is a difference between the subjective questionnaire and the physiological index measurement. Eye movement experiments imply that the illumination of the lamp and the color of the wall will cause changes in the physiological indicators and visual comfort of the customer; the questionnaire data proves that the illumination of the lamp, the color of the wall and the style of decoration will affect the visual comfort of the consumer. This is consistent with the findings of Warakul Tantanatewin and Dongyoup Kimet (Warakul Tantanatewin and Vorapat Inkarojrit ,2018; Dongyoup Kim et al., 2020). At the same time, the results of eye movement experiments and the study of Elliot et al.(2012) concluded that individuals cannot control emotional and physiological responses to color, but indoor lighting can affect emotional and physiological function, and pupil diameter and number of fixations can be used to measure visual comfort is consistent(Knez et al., 2010; Vytautas Abromavičius and Artūras Serackis, 2018), validating the scientific rationale for the introduction of physiological index measurement methods (Knez and Hygge, 2010). Therefore, this paper introduces visual comfort from the perspective of emotion, and adopts the method of combining subjective and objective to measure emotion, which makes up for the deficiency of the existing subjective cognitive perspective.

Eye-tracking data concluded that the illuminance of the light fixture and the color of the walls have significant impact on visual comfort. Lighting intensity and music style have interactive effects. The conclusion from the questionnaire data is that the illuminance of lamps, the color of the wall, and the decoration style have significant impact on visual comfort. There is no interaction between light levels and musical styles. There are two reasons for the difference between the two measurement methods.

(1) Elliot and Maier(2012) found that people’s reactions to colors are always unconscious, and people cannot control their emotional and physiological reactions to colors(Elliot, 2012). Sebba’s research confirmed that there was a structural correspondence between color and music(Sebba, 1991). Because this response is involuntary and evokes a relatively small degree of mood change, subjective judgment alone cannot accurately measure the subject’s true mood change. But this change can be clearly reflected in eye movement indicators. (2) According to the “emotion-cognitive-motor response” model proposed by Izard, emotion is the basic motivation, and cognition plays an important role. The integration of emotional, cognitive, and motor systems produced certain experiences, emotions, and responses(Izard, 1977). Compared with the illuminance of lamps and the color of walls, personal perception of decoration style is acquired through acquired learning, and there is a more complex cognitive judgment process. Among them, higher cognitive functions such as perception and memory retrieval play a more important role in the perception process. Therefore, the differences in participants’ responses to different decoration styles are more influenced by their previous experiences and preferences. This difference is not obvious in the eye movement index, but it is more obvious in the subjective judgment. This also proves the necessity of combining subjective questionnaires with objective physiological indicators.

The results of the study confirmed the interaction between illuminance, wall color and decoration style, which are consistent with the results of Babin et al.(2003). There is an interaction between light and color, light will weaken the color effect, any color and the negative effects of both can be overcome by introducing other factors that can enhance evaluation and excitement(Babin et al.,2003). But there is no interaction between decorative style and musical style. There is no significant difference between European-style hotels playing Chinese or European-style music. This differs from the conclusion of Demoulin (2011), who argued that music consistent with the service environment could create a more comfortable atmosphere(Demoulin,2011). Regarding the differences in the research results, on the one hand, the subjects themselves may have no obvious preference for Chinese-style or European-style music. On the other hand, consumers spend less time in the hotel lobby than in the restaurant waiting environment, so the music style reflects insignificance.

**3.Objective eye movement indicators and subjective assessment of visual comfort and mood were employed, however, I did miss the.**

**Response:** Thank you very much for your comment. In this study, objective eye movement indicators and subjective comfort, mood questionnaires are used to assess visual comfort and mood. In the results part of the paper, the subjective and objective data are analyzed concretely. The eye movement data is analyzed by two indicators, pupil diameter and fixation points. It is concluded that the illumination of lamps and the color of walls have significant impacts on visual comfort. Light intensity and music style have interactive effects. The conclusions of the questionnaire data that includes the illumination of the lamp, the color of the wall, and the decoration style have significant effect on the visual comfort. There is no interaction between light illuminance and musical style.

**4.The author explained the effects of decoration style with the prior experiences of participants? As I mention in previous comments, were the experience not pre-assessed or added it as covariate? Please also added it as the limitation.**

**Response:** Thank you very much for your comments. The subjects of the pre-experiment have experience of staying in Chinese-style and European-style hotels, the subjects of the formal experiment have the experience of staying in ordinary hotels, but have no experience of staying in Chinese-style or European-style hotels. They have no apparent preference for style. We have added this part to the participants module in the text.

In the discussion part, we use the participants’ previous experience to explain the influence of the decoration style. The experience here does not refer to their experience of staying in a Chinese-style hotel or a European-style hotel, but the participants’ perception of these two styles. What we explain here is why the eye-tracking data concluded that the effect of decoration style on visual comfort is not significant, while the results obtained from the subjective questionnaire is significant. Because compared with the illuminance of lamps and the color of walls, personal perception of decoration style is acquired through acquired learning, and there is a more complex cognitive judgment process. Among them, higher cognitive functions such as perception and memory retrieval play a more important role in the perception process. Therefore, the differences in participants’ responses to different decoration styles are more influenced by their previous experiences and preferences. This difference is not obvious in the eye movement index, but it is more obvious in the subjective judgment.

**5.The current study showed much significance in practical sensory, market of hotel as well as in theoretical work. Please state it more concise. In addition, the writing expression need to be improved throughout the draft, please ask one native speaker of English to polish the writing before re-submit.**

**Response:** Thank you very much for your advice.

We have succinctly sorted out the implications of the current research for practical sensory, hotel market and theoretical work.

We have sent this article to a native English-speaking professional for proofreading and have revised according to the professional advice.

**Response to Reviewer #2:**

Thank you very much for the insightful comments on our paper. We have revised our paper carefully according to these suggestions. In this note, we will first list your comments and then outline what we have done in response to these comments.

**1.Experimental studies, color, light, music, etc. Studying many factors together affects the validity of the experiment. Therefore, there is a problem with the limitations of the research.**

Thank you very much for your comment. The effects of lighting on mood and comfort, the effects of color on mood and comfort, and the effects of music on mood and comfort are all well-researched.

(1)Color, emotion and visual comfort in service setting

Color is one of the key visual dimensions of the environment that has an impact on behavior and emotions. The emotional effect of color was particularly important in the hospitality industry because emotions comprise a powerful affective component of customer satisfaction. The differences between two contrast hues( blue and red) with two levels for saturation and brightness were examined in an experimental study. The results indicated participants enjoyed staying in blue hotel rooms more than in the red ones. The blue room was more pleasant, comfortable, and relaxing(Katsiaryna Siamionava et al., 2018). In the hotelscape, color had been shown to significantly impact customers’ aesthetic perceptions (Alfakhri et al., 2018). A comparison of warm and cool colors revealed that cool colors were not aesthetically superior to warm colors in the luxury hotel context and warmer colors were more pleasing(Warakul Tantanatewin and Vorapat Inkarojrit, 2018; Dongyoup Kim et al., 2020). However, an academic study also demonstrated that no statistically significant effects of color and temperature on comfort results within the tested settings(Arianna Latini et al., 2021).

(2)Light illuminance, emotion and visual comfort in service setting

Lighting was an indispensable factor in the design of the hotel lobby. Lighting affected the overall impression and perceived comfort of the lobby(Akita, 2019; Countryman and Jang, 2006). The level of visual comfort in lighting in a residential context turned to be more influenced by the color temperature and illuminance compared to other factors(Yoon et al., 2014). Under high illumination, the level of anxiety would decrease, which was more conducive to relieving fatigue. Providing a user-friendly and user-controlled lighting system for hotel rooms helped improve customer satisfaction(Akita, 2019). Light not only affected people’s emotions, but the interaction between light and sound also affected people’s collection of target information. Low-illumination light was more likely to cause visual fatigue. Lower contrast colors with darker lighting and simple decoration made it easier to create a relaxed and romantic atmosphere. Dim light with soothing music can create a relaxed and comfortable atmosphere(Siamionava et al., 2018). A lower color temperature would create a higher sense of security, positive feelings and restfulness(Akita, 2019). While some scholars found that brighter lights were more conducive to positive emotions( Figueiro, 2016). Different environments have different lighting requirements.

(3)Color, light illuminance, music, emotion and visual comfort in service setting

There were interactions between the senses, the interaction between senses was mainly reflected in their direct impact on emotions( Kantonoet et al., 2019). The tone stimulus can alter the pleasantness rating of stimuli from other senses(Dematte`et al., 2007; Pollatos et al. 2007; Logeswaran and Bhattacharya, 2009). For example, congruent sounds can enhance odor pleasantness to a higher degree than can incongruent sounds(Seo H S, 2011). Music generally interacted with other environmental factors and music emotion was significantly correlated with lighting(Morin et al., 2007; Hsiao et al., 2017). There were also structural correspondences between color and music(such as emotional expression, hierarchical organization and contrast)(Sebba,1991). Demoulin(2011) studied the consistency of music and the overall atmosphere, and the results showed that when coordinated with the service environment and music, the relaxing, calm and pleasant atmosphere would be created(Demoulin, 2011).

Although music is not a visual factor, it will have an impact on the visual comfort of lighting, color and style. There is an interaction between these four factors. What we see in real life is the overall comfort of the hotel lobby, not the comfort brought by individual lights or colors. Therefore, these four factors are inseparable, and we put them in a model to study.

**2.In the second study, although the sample size seems to be sufficient in an experimental study, the design of the experiment as a control group and a subject group is not clear. What kind of experiment did the authors do? The explanation of the method proving the validity of the experiment could not be understood.**

**Response:** Thank you very much for your comments. The eye-tracking experiment is a within-subject experiment. According to the three variables of 3 (light illumination: bright illumination, normal illumination, dim) illumination × 2 (wall color: cool color, warm color) × 2 (decoration style: European style, Chinese style), we used 3d max software to make 12 3D lobby models. Eye-tracking data and questionnaire data were collected by subjects viewing a hotel lobby model.

Each subject went through 5 groups of experiments, with a 10-minute rest between each group of experiments.

In the first group, under no music background, the subjects observed the 12 hotel lobby models (The first group of experiments collected two parts of data. 1. Collecting eye movement data, 12 hotel lobby models appeared randomly, and eye trackers captured the eye movement data. 2. Collecting the questionnaire data, and the subjects will score on the questionnaire after watching the model).

The second group, under the background of European-style music, the subjects observed the European-style hotel lobby (The second group of experiments collected two parts of data. 1. Collecting eye movement data, 6 hotel lobby models appeared randomly, and the eye tracker captured the eye movement data. 2. Collecting the questionnaire data, and the subjects will score on the questionnaire after watching the model).

The third group, under the background of European music, the subjects observed the Chinese-style hotel lobby (the third group of experiments collected two parts of data. 1. Collecting eye movement data, 6 hotel lobby models appeared randomly, and the eye tracker captured the eye movement data. 2. The questionnaire data was collected, and the subjects scored on the questionnaire after watching the model).

The fourth group, under the background of Chinese music, the subjects observed the Chinese hotel lobby (the fourth group of experiments collected two parts of data. 1. Collecting eye movement data, 6 hotel lobby models appeared randomly, and the eye tracker captured the eye movement data. 2. The questionnaire data was collected, and the subjects scored on the questionnaire after watching the model).

The fifth group, under the background of Chinese music, the subjects observed the lobby of a European-style hotel (the fifth group of experiments collected two parts of data. 1. Collecting eye movement data, 6 hotel lobby models appeared randomly, and the eye tracker captured the eye movement data. 2. The questionnaire data was collected, and the subjects scored on the questionnaire after watching the model).

Fig.1. Flow chart of experimental stimulus presentation

**3.Brightness and warm-cold tones are emphasized in the use of colors and lights. However, colors must be expressed by giving technical scale ranges. Expressing it as hot-cold is insufficient. Regarding the light, the degree of brightness should also be shown by giving the value of the lamps used.**

**Response:** Thank you very much for your comments. The specific technical data of the color are listed in Table 1 in the text. Bright light illumination is 13000cd, normal light illumination is 9000cd, dim light illumination is 2000cd. The data are in the Apparatus and stimuli section of the paper, please check.

Table 1. Color indicators of room models.

| Serial Number | Variable | Hue | Saturation | Value | Sample | RGB | Color Number |
| --- | --- | --- | --- | --- | --- | --- | --- |
| 1 | Cool color | 26° | 0 | 0 |  | R：255  G：255  B：255 | #ffffff |
| 2 | Warm color | 26° | 34 | 19 |  | R：223  G：181  B：148 | #dfb594 |

Note.R is the intensity value of the red channel. G is the intensity value of the green channel. B is the intensity value of the blue channel.

**4.Although the combination of many factors causes the complexity of the boundaries of the research, if the above deficiencies are completed in the research, an understandable situation will occur.**

**Response:** Thanks a lot for your comment. The effects of lighting on mood and comfort, the effects of color on mood and comfort, and the effects of music on mood and comfort are all well-researched, as we described in our answer to question 1. Music is not a visual factor, but it can have an impact on visual comfort with lighting, color and style. There is an interaction between these four factors. What we see in real life is the overall comfort of a hotel lobby, not the comfort of individual lights or colors. Therefore, these four factors are inseparable, and we put them together in a model to study.

**5.The sources used by the authors are quite old. In particular, there are many specific journals in this field such as Color&Research. And in these journals, there are color and light studies with high scientific value. The bibliography can be enriched.**

**Response:** Thank you very much for your advice. Part of the older literature in this study is the definition of comfort and the introduction of human physiology. References have been updated and enriched wherever possible, based on journal articles such as Color & Research. Please check it.
