# Supplementary material for: Analysis of factors affecting visual comfort in hotel lobby
Source: PLoS One. 2023 Jan 19;18(1):e0280398. doi: 10.1371/journal.pone.0280398 (PMC9851505; doi:10.1371/journal.pone.0280398)
Supplement: Attachment — Submitted filename: Response to Reviewers.doc [file pone.0280398.s005.doc]

**Response to Reviewer #1:**

Thank you again very much for the insightful comments on our paper. We have revised our paper carefully according to these suggestions. In this note, we will first list your comments and then outline what we have done in response to these comments.

1) **The reational of the study combining multiple factors together in a study as each of the factor had influence on visual comfort.**

**Response:** Thank you very much for your comment. The effects of lighting on mood and comfort, the effects of color on mood and comfort, and the effects of music on mood and comfort are all well-researched.

(1)Color, emotion and visual comfort in service setting

Color is one of the key visual dimensions of the environment that has an impact on behavior and emotions. The emotional effect of color is particularly important in the hospitality industry because emotions comprise a powerful affective component of customer satisfaction. The differences between two contrast hues( blue and red) with two levels for saturation and brightness were examined in an experimental study. The results indicated participants enjoyed staying in blue hotel rooms more than in the red ones. The blue room was more pleasant, comfortable, and relaxing(Katsiaryna Siamionava et al., 2018). In the hotelscape, color had been shown to significantly impact customers’ aesthetic perceptions (Alfakhri et al., 2018). A comparison of warm and cool colors revealed that cool colors were not aesthetically superior to warm colors in the luxury hotel context and warmer colors were more pleasing(Warakul Tantanatewin and Vorapat Inkarojrit, 2018; Dongyoup Kim et al., 2020). However, an academic study also demonstrated that no statistically significant effects of color and temperature on comfort results within the tested settings(Arianna Latini et al., 2021).

(2)Light illuminance, emotion and visual comfort in service setting

Lighting was an indispensable factor in the design of the hotel lobby. Lighting affected the overall impression and perceived comfort of the lobby(Akita, 2019; Countryman and Jang, 2006). The level of visual comfort in lighting in a residential context turned to be more influenced by the color temperature and illuminance compared to other factors(Yoon et al., 2014). Under high illumination, the level of anxiety would decrease, which was more conducive to relieving fatigue. Providing a user-friendly and user-controlled lighting system for hotel rooms helped improve customer satisfaction(Akita, 2019). Light not only affected people’s emotions, but the interaction between light and sound also affected people’s collection of target information. Low-illumination light was more likely to cause visual fatigue. Lower contrast colors with darker lighting and simple decoration made it easier to create a relaxed and romantic atmosphere. Dim light with soothing music could create a relaxed and comfortable atmosphere(Siamionava et al., 2018). A lower color temperature would create a higher sense of security, positive feelings and restfulness(Akita, 2019). While some scholars found that brighter lights were more conducive to positive emotions( Figueiro, 2016). Different environments have different lighting requirements.

(3)Color, light illuminance, music, emotion and visual comfort in service setting

There were interactions between the senses, the interaction between senses was mainly reflected in their direct impact on emotions( Kantonoet et al., 2019). The tone stimulus could alter the pleasantness rating of stimuli from other senses(Dematte et al., 2007; Pollatos et al., 2007; Logeswaran and Bhattacharya, 2009). For example, congruent sounds could enhance odor pleasantness to a higher degree than can incongruent sounds(Seo, 2011). Music generally interacted with other environmental factors and music emotion was significantly correlated with lighting(Morin et al., 2007; Hsiao et al., 2017). There were also structural correspondences between color and music(such as emotional expression, hierarchical organization and contrast)(Sebba,1991). Demoulin(2011) studied the consistency of music and the overall atmosphere, and the results showed that when coordinated with the service environment and music, the relaxing, calm and pleasant atmosphere would be created(Demoulin, 2011).

Although music is not a visual factor, it will have an impact on the visual comfort of lighting, color and style. What we see in real life is the overall comfort of the hotel lobby, not the comfort brought by individual lights or colors. Therefore, these four factors are inseparable, and we put them in a model to study.The results of the study by Barry J. Babin et al. (2003) showed that the results change considerably when the effects of lighting in combination with color was taken into account. The experimental results proved that there was indeed an interaction between lamp illumination, wall tone and decoration style(Babin et al., 2003).

2) **The 3D pictures were employed to exposure different visual elements to participants, however the presented pictures seems not definitely reflect the manipulation of the factors such as color, and also several pictures with such higher brightness or darknee that they were difficult to see clear, all these would limite the possible experiences of participants.**

**Response:** Thank you very much for your comment. A 3D hotel lobby model was based on a compilation of typical hotel lobby. The lobby consisted of the basic attributes of a typical hotel lobby: waiters, decorative lights, and front desk cabinets. Therefore, the models did not definitely reflected the manipulation of color and other factors, but the model tried to keep other factors consistent, except for the light intensity, wall tone and decoration style.

The specific intensity of light illumination was determined according to the opinions of pre experiment experts and subjects. The reason why some pictures had high or low brightness was that there were three levels of light illumination: bright illumination, normal illumination and dim illumination. The experimental results showed that the illumination comfort of bright light was relatively high, while that of dim light was the lowest.

3) **The 2*2*2*2*3 within subjects design was employed leading a quiet complexy analysis and the interaction effects would be meaningless.**

**Response:** Thank you very much for your comment. In reality, consumer comfort is determined by the environment after the interaction of multiple factors, rather than a single factor. Lighting, color and decoration style are are inseparable in our visual perception. Multisensory interaction studies and multifactor interaction studies are increasing. We refer to multifactor interaction studies from leading journals in various fields. For example, in the field of sensory marketing, there were many papers that studied the repetitive experiments within subjects with multiple factors and studied the impact of interaction, such as Wonyoung Yanga and Hyeun Jun Moon(2019)^[1]^ investigated the influence of multisensory interaction on acoustic comfort, thermal comfort, visual comfort, and indoor environmental comfort. Their experimental design was 3(Homogenous room temperatures: 20, 25, and 30 °C )×3(Illuminance levels: 150,500, and 1000 lx) × 4( Different types of sound : babble, fan, music, and water) × 4(Sound levels: 45, 55, 65, and 75 dBA) (Yang W and Moon HJ, 2019).

Katsialyna Siamonava et al^[2]^. (2018) studied the impact of space color on guests’ perception of hotel rooms, and they adopted 2c×2×2 design . The results showed that the three interactions were not significant, but at the 1% confidence level, two of the two interactions were significant(Katsialyna Siamonava et al., 2018).

Holger Roschk et al.^[3]^(2017) examined the effects of music(presence vs. absence), scent (presence vs. absence), and colors (warm: red, orange, and yellow vs. cool: green, blue, violet,and white) on shopping outcomes. The results showed that being in an environment with music or scents produced higher ratings of pleasure, satisfaction, and behavioral intention compared to environments without these conditions(Holger Roschk et al., 2017).

The study design by Barry J. Babin et al.^[4]^(2003) was 2(color: blue, orange) × 2(light: bright, soft) ×2 (price: high price, low price). The results of the ANOVA and post hoc tests indicated an interaction between the three, the results changed substantially when the effect of lighting in combination with color was considered. The use of soft lights with an orange interior generally nullified the ill effects of orange and produced the highest level of perceived price fairness while controlling for price(Babin et al., 2003).

4) **The sample size was relatively small.**

**Response:** Thank you very much for your comment. The sample size of 43 people is indeed relatively small, but it also meets the requirement of the number of people in the consumer eye movement experiment. The proof that we have the required number of subjects is as follows, for example, Peter Walla et al.(2011) used an eye-tracking experiment to study emotional and physiological responses to brand attitudes in 29 subjects (Peter Walla et al., 2011). Qiuzhen Wang et al. (2014) studied eye-tracking of website complexity from a cognitive load perspective, with 42 college students as subjects(Qiuzhen Wang et al., 2014). Borisuit et al.(2014) showed that workplace lighting conditions affected various factors related to job satisfaction, productivity, and well-being. Lighting conditions affected visual comfort, mood, alertness, and well-being, 25 People were tested(Borisuitet et al. 2014). Research by Vytautas Abromavičius and Artūras Serackis(2018) showed that pupil diameter and the number of fixation points can be used to measure visual comfort. The “uncomfortable” visual comfort score had the highest pupil size and lower fixation points. There were 24 subjects in the eye movement experiment(Vytautas Abromavičius and Artūras Serackis, 2018). Cajochen et al.(2019) studied that lighting conditions affected subjects’ visual comfort, mood, and sleep intensity. The number of subjects was 15(Cajochen, 2019). Yu Wang et al.(2020) investigated the interactive effects of illumination and the associated color temperature on observers’ color preference and degree of white light perception with 30 subjects(Yu Wang et al., 2020). Arianna Latini et al.(2021) used immersive virtual reality to assess productivity and comfort in the workplace with 23 subjects(Arianna Latini et al., 2021).

5) **The analysis need to be improved and the explanation also need to be expand. In addition, the The readability of the most sentences still need to be polish.**

**Response:** Thank you very much for your advice. We have added new interaction charts and refined the analysis and interpretation of the data. Babin et al. (2003) applied ANOVA and post hoc tests to verify consumer responses to various combinations of color, lighting, and price point(Babin et al., 2003). Han-Seok Seo et al.(2011) used ANOVA and post hoc tests to verify the integration effect of auditory and olfactory sensations(Han-Seok Seo et al., 2011). Katsiaryna Siamionava et al.(2018) used ANOVA to study the effect of colors of different hue, saturation and brightness on consumers(Katsiaryna Siamionava et al., 2018).

Our data analysis method was to apply the aov function in the R language to do repeated ANOVA and post hoc tests.

We sent this article to another native English speaker for proofreading, and revised it according to professional suggestions.

**Response to Reviewer #2:**

Thank you again very much for the insightful comment on our paper. We sent this article to another native English speaker to proofread and fix the typos and other issues.

1. Yang W, Moon HJ. Combined effects of acoustic, thermal, and illumination conditions on the comfort of discrete senses and overall indoor environment. Building and Environment. 2019.
2. Siamionava K, Slevitch L, Tomas SR. Effects of spatial colors on guests’ perceptions of a hotel room. International Journal of Hospitality Management. 2018;70:85-94.
3. Roschk H , Loureiro S M C , Breitsohl J . Calibrating 30 Years of Experimental Research: A Meta-Analysis of the Atmospheric Effects of Music, Scent, and Color[J]. Journal of Retailing, 2017(2):228-240.
4. Babin BJ, Hardesty DM, Suter TA. Color and shopping intentions. Journal of Business Research. 2003;56(7):541-51.
