# Supplementary material for: Analysis of factors affecting visual comfort in hotel lobby
Source: PLoS One. 2023 Jan 19;18(1):e0280398. doi: 10.1371/journal.pone.0280398 (PMC9851505; doi:10.1371/journal.pone.0280398)
Supplement: Attachment — Submitted filename: Response to Reviewer.doc [file pone.0280398.s006.doc]

**Response to Reviewer :**

Thank you again very much for the insightful comments on our paper. We have revised our paper carefully according to these suggestions. In this note, we will first list your comments and then outline what we have done in response to these comments.

1) **Although I completely understand why you picked up the term - why don’t you use “visual setting” or “conformation”. Please clarify with a theoretical perspective, there is no need to emphasise past studies and the “common term”.**

**Response:** Thank you very much for your advice. There were many studies on “visual setting” or “conformation”, such as Katsiaryna Siamionava et al. (2018) showed that wall color in visual setting affected consumer perception. Dongyoup Kim et al. (2020) showed that the visual service setting in luxury hotels affected the behavior and emotions of the subjects. After summarizing the factors that affected hotel comfort, we found that visual setting affects consumer perception and played the most important role in sensory evaluation factors. However, visual comfort, which ultimately affected people’s motivation to consume and how visual setting affected visual comfort, was often overlooked.

Light illumination, wall tones, and decorative style in the visual setting are the independent variables in this study. Our main objective is to investigate their effects on visual comfort and to explore the visual environment in which consumers’ visual comfort would be higher. We discuss the reasons for choosing visual comfort as the dependent variable in the paper, please check. Past relevant studies and definitions of visual comfort were the theoretical basis for our choice of eye movement metrics. If you feel that they are not needed for this study, we can remove them.

2) **The authors mentioned about colour processing (and perception, per se) - so then I ask the authors to check references on confounding factors. For example, were excluded if had any substance use (references), use of meds (references) and so it goes.**

**Response:** Thank you very much for your advice. Based on your suggestions, we have combed through the color-related literature de novo, categorized and summarized the existence of color interactions as well as non-interactions, and explained the reasons why we chose to study color-light interactions.

3) **I still think ANOVA isn’t correct. I understand that you pointed out some refs that run ANOVA, but you have more than one DV. Seems really different from a 2 x 2 … Please consider MANOVA, effect sizes and CIs.**

**Response:** Thank you very much for your advice. Based on your advice, we have re-analyzed the data using MANOVA, please check.

4) **Please place limitations and further directions for researchers and readers on a smoother way (for example, you have some limitations here and there, but others need to interpret properly and, if want to, replicate basing on your arguments).**

**Response:** Thank you very much for your advice. We have freshly combed this section of the article on limitations to provide further guidance for researchers and readers.

**5) Check the references that are not in the text.**

**Response:** Thank you very much for your comment.We have checked all references and updated some of them, please check them.
